# The effects of selected inhibitors on human fetal adrenal steroidogenesis differs under basal and ACTH-stimulated conditions

**DOI:** 10.1186/s12916-021-02080-8

**Published:** 2021-09-08

**Authors:** Cecilie Melau, Malene Lundgaard Riis, John E. Nielsen, Signe Perlman, Lene Lundvall, Lea Langhoff Thuesen, Kristine Juul Hare, Mette Schou Hammerum, Rod T. Mitchell, Hanne Frederiksen, Anders Juul, Anne Jørgensen

**Affiliations:** 1 Department of Growth and Reproduction, Copenhagen University Hospital – Rigshospitalet, Blegdamsvej 9, DK-2100 Copenhagen, Denmark; 2 International Center for Research and Research Training in Endocrine Disruption of Male Reproduction and Child Health, Copenhagen University Hospital – Rigshospitalet, Copenhagen, Denmark; 3 Department of Gynaecology, Copenhagen University Hospital – Rigshospitalet, Copenhagen, Denmark; 4 Department of Obstetrics and Gynaecology, Copenhagen University Hospital - Hvidovre and Amager Hospital, Hvidovre, Denmark; 5 Department of Obstetrics and Gynaecology, Copenhagen University Hospital - Herlev and Gentofte Hospital, Herlev, Denmark; 6 MRC Centre for Reproductive Health, The Queen’s Medical Research Institute, The University of Edinburgh, Edinburgh, UK; 7 Department of Clinical Medicine, University of Copenhagen, Copenhagen, Denmark

**Keywords:** Human, Adrenal, *Ex vivo*, Steroid hormones, ACTH, Osilodrostat, Efavirenz, Abiraterone

## Abstract

**Background:**

Disordered fetal adrenal steroidogenesis can cause marked clinical effects including virilization of female fetuses. In postnatal life, adrenal disorders can be life-threatening due to the risk of adrenal crisis and must be carefully managed. However, testing explicit adrenal steroidogenic inhibitory effects of therapeutic drugs is challenging due to species-specific characteristics, and particularly the impact of adrenocorticotropic hormone (ACTH) stimulation on drugs targeting steroidogenesis has not previously been examined in human adrenal tissue. Therefore, this study aimed to examine the effects of selected steroidogenic inhibitors on human fetal adrenal (HFA) steroid hormone production under basal and ACTH-stimulated conditions.

**Methods:**

This study used an established HFA *ex vivo* culture model to examine treatment effects in 78 adrenals from 50 human fetuses (gestational weeks 8–12). Inhibitors were selected to affect enzymes critical for different steps in classic adrenal steroidogenic pathways, including CYP17A1 (Abiraterone acetate), CYP11B1/2 (Osilodrostat), and a suggested CYP21A2 inhibitor (Efavirenz). Treatment effects were examined under basal and ACTH-stimulated conditions in tissue from the same fetus and determined by quantifying the secretion of adrenal steroids in the culture media using liquid chromatography-tandem mass spectrometry. Statistical analysis was performed on ln-transformed data using one-way ANOVA for repeated measures followed by Tukey’s multiple comparisons test.

**Results:**

Treatment with Abiraterone acetate and Osilodrostat resulted in potent inhibition of CYP17A1 and CYP11B1/2, respectively, while treatment with Efavirenz reduced testosterone secretion under basal conditions. ACTH-stimulation affected the inhibitory effects of all investigated drugs. Thus, treatment effects of Abiraterone acetate were more pronounced under stimulated conditions, while Efavirenz treatment caused a non-specific inhibition on steroidogenesis. ACTH-stimulation prevented the Osilodrostat-mediated CYP11B1 inhibition observed under basal conditions.

**Conclusions:**

Our results show that the effects of steroidogenic inhibitors differ under basal and ACTH-stimulated conditions in the HFA *ex vivo* culture model. This could suggest that in vivo effects of therapeutic drugs targeting steroidogenesis may vary in conditions where patients have suppressed or high ACTH levels, respectively. This study further demonstrates that *ex vivo* cultured HFAs can be used to evaluate steroidogenic inhibitors and thereby provide novel information about the local effects of existing and emerging drugs that targets steroidogenesis.

**Supplementary Information:**

The online version contains supplementary material available at 10.1186/s12916-021-02080-8.

## Background

Human adrenals secrete mineralocorticoids, glucocorticoids, and androgens from early fetal life [1–4]. Thus, adrenals are active endocrine glands prior to the formation of the cortex and medulla, which represent the endocrine secreting zones in the adult gland. The lifelong secretion of adrenal steroid hormones is critical in regulating salt levels, metabolism, and the immune system [5]. Accordingly, imbalanced adrenal steroidogenesis can lead to various disorders resulting from both reduced and excess production of adrenal steroids, including adrenal insufficiency and Cushing syndrome (CS), respectively [6, 7]. Hence, mutations causing excess levels of adrenal androgens may manifest during development as described in virilized female fetuses with congenital adrenal hyperplasia (CAH), frequently caused by deficient 21-hydroxylase activity. If not treated, classic CAH may cause a life-threatening postnatal salt-wasting condition as described in neonates of both sexes [8]. Treatment of imbalanced steroid hormone levels depends on the cause of the disease but can involve hormone replacement therapy and medication targeting excess steroid hormone production. In addition to the therapies used to specifically target adrenal disorders, therapeutic drugs used for other indications can have undesired side-effects on adrenal function, including Efavirenz which is used for treatment of HIV but has been suspected to also inhibit adrenal steroidogenesis [9].

In addition to the well-described classic biosynthesis of adrenal steroid hormones, the importance of adrenal synthesis of alternative (also described as the “backdoor pathway”) and 11-oxygenated adrenal androgens has gained attention in recent years, which has challenged the understanding of adrenal endocrine function in both health and disease. Thus, enzymes involved in the alternative pathways have also been associated with differences/disorders of sex development, thereby explaining some of the observed phenotypes that did not fit with the understanding based solely on information about the classic adrenal steroidogenesis [6]. Enzymes involved in androgen synthesis via the alternative pathway have been detected in human fetal adrenals (HFA) [10] thereby emphasizing that many unresolved questions remain about the regulation and function of human fetal adrenal steroidogenesis in health and disease.

Classic adrenal steroidogenesis is primarily regulated through the action of adrenocorticotropic hormone (ACTH), which induces the biosynthesis of cortisol and androgens in vivo. Imbalanced adrenal steroidogenesis can originate from mutations causing disrupted steroid enzyme functions and/or dysregulated secretion of ACTH [11]. ACTH levels are regulated by the hypothalamic-pituitary-adrenal (HPA) axis and secretion of adrenal cortisol acts as a negative feedback loop. The HPA axis plays an important role in regulating adrenal endocrine function throughout fetal and adult life [12, 13] and can be both suppressed and elevated in adrenal disorders highlighting the importance of understanding effects of steroidogenic drugs under normal, deficient, and ACTH-stimulated conditions.

Importantly, the morphological and functional differences in adrenal glands between humans and the majority of animals [14–16] have reduced the applicability of testing steroidogenic drugs affecting adrenal steroidogenesis in animal models. However, our recently established HFA *ex vivo* culture model provides a model to examine de novo steroid hormone production and manipulated steroidogenic activity under basal and ACTH-stimulated conditions [17]. Thus, this study aimed to examine the effects of the CYP17A1 inhibitor Abiraterone acetate, the CYP11B1/2 inhibitor Osilodrostat, and the proposed CYP21A2 inhibitor Efavirenz [9] on HFA tissue cultured *ex vivo.* Changes in classic adrenal steroid secretion following inhibitor treatment were investigated under basal and ACTH-stimulated conditions, and effects on HFA tissue expression of steroidogenic enzymes were examined.

## Methods

**Fig. 1:**
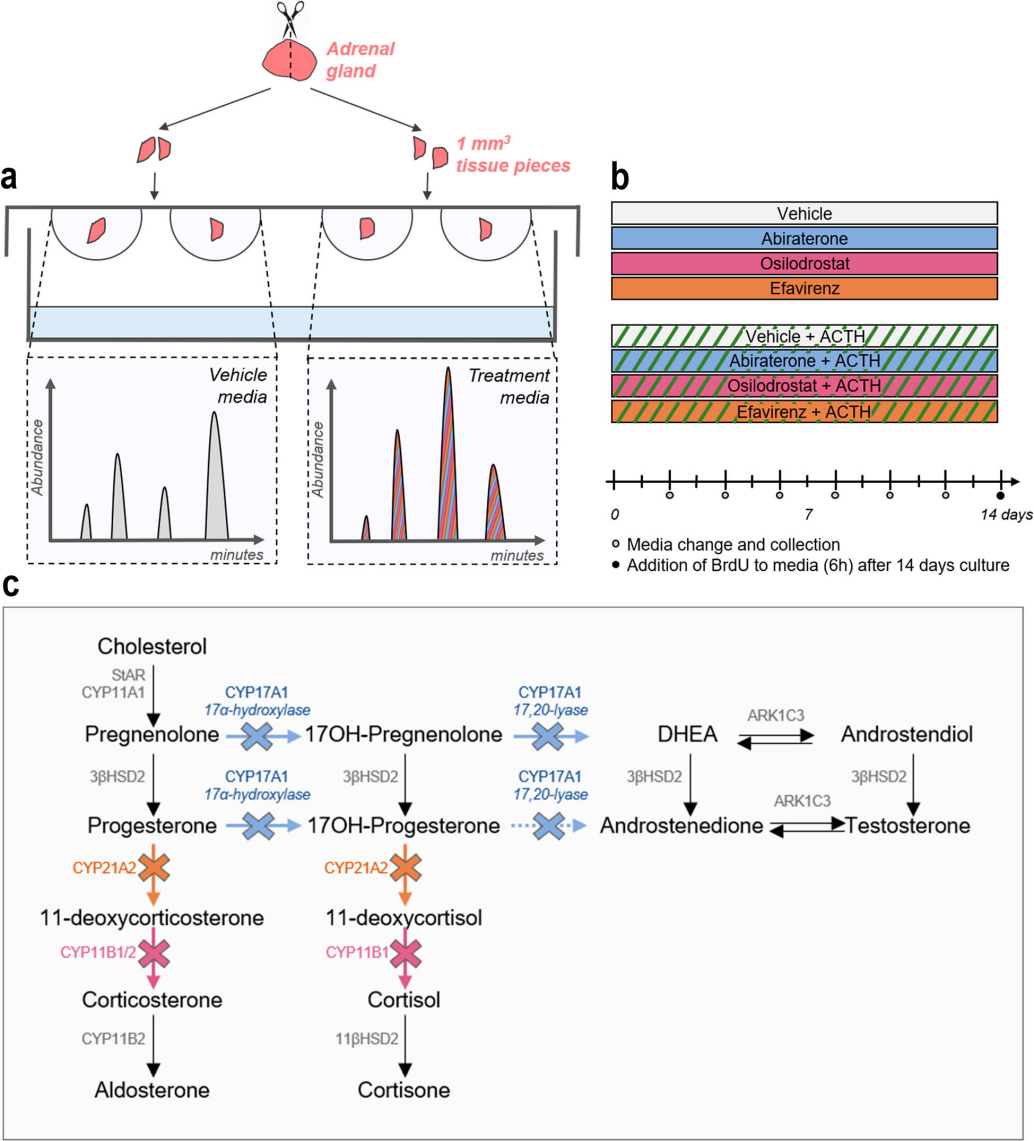
Experimental overview of human fetal adrenal *ex vivo* culture approach and the selected inhibitors. **a** Illustration of the hanging drop culture approach and measurements of steroid hormones by LC-MS/MS in the media. **b** Overview of media collection days and treatment setups under basal and ACTH-stimulated conditions. Media were pooled from all technical replicates collected throughout the experimental period of 14 days. **c** Overview of classic adrenal steroidogenesis. Arrows indicate enzyme reactions; stippled arrow indicates enzyme reaction with low substrate affinity. Colored crosses indicate enzymes expected to be inhibited by the selected inhibitors. Blue: Abiraterone acetate, magenta: Osilodrostat, orange: Efavirenz

The effects of three selected steroidogenic inhibitors on HFA steroidogenesis was examined under basal and ACTH-stimulated conditions in an established *ex vivo tissue* culture model. HFA tissue fragments were cultured for 14 days with Abiraterone acetate, Osilodrostat, or Efavirenz supplemented to the culture media. Since the inhibitory effects were examined under basal and ACTH-stimulated conditions, both adrenals from the same fetus were used (Fig. 1a, b). The main endpoint was quantification of classic steroid hormone metabolites, but tissue expression of selected steroidogenic enzymes as well as proliferation and apoptosis markers were also examined. The inhibitors were chosen based on their reported steroidogenic enzyme target in the classic adrenal steroidogenic pathway. Hence, Abiraterone acetate inhibits 17α-hydroxylase and 17,20-lyase activities of CYP17A1, Osilodrostat inhibits the 11β-hydroxylase activity of CYP11B1/2 and the aldosterone synthase activity of CYP11B2, while Efavirenz has been suggested to inhibit CYP21A2 [9] (Fig. 1c).

### Tissue collection

Fetal tissue (gestational weeks (GW) 8–12) used in this study was available following elective surgical termination of pregnancy at Copenhagen University Hospital (Rigshospitalet), Hvidovre Hospital, and Herlev Hospital. The Danish regional ethics committee (permit number H-1-2012-007) approved the collection and use of fetal material in this study. Medical staff working independently of the project recruited all participating women who gave their informed written and oral consent. None of the terminations were for reasons of fetal abnormality or pathology of pregnancy. Fetal material was kept at 4 °C immediately after termination of pregnancy and during transport to the laboratory. Fetal age was determined by scanning crown-rump length and by evaluation of foot length [18]. Fetal adrenal tissue was dissected in ice-cold PBS and immediately setup in *ex vivo* cultures. Both adrenals from the same fetus were used in experimental setups examining treatment effects under both basal and ACTH-stimulated conditions, while one adrenal was used when examining treatment effects under basal conditions only. Thus, in total 78 adrenals were used for *ex vivo* cultures corresponding to adrenals from 50 individual fetuses.

### *Ex vivo* tissue culture setup

HFAs were cultured *ex vivo* as 1 mm^3^ tissue fragments*,* as previously described using a hanging drop culture approach [17]. To ensure an equal representation of HFA morphology in the vehicle vs. treatment setup, adrenal glands were initially divided into two halves and subsequently further divided into fragments (Fig. 1a). To ensure an equal allocation into the initial halves this was done using a dissection microscope (Nikon SMZ800N, Japan) on top of Millimeter graph paper (1 mm^2^ boxes). Afterward, all pieces from one half of the gland were cultured in vehicle-control media, and all pieces from the other half in treatment media corresponding to 1–13 fragments per treatment depending on the initial size of the HFA. Tissue fragments were cultured in 40 μl media for 14 days at 37 °C under 5% CO_2_ with a complete media change every 48 h. Media were collected throughout the entire culture period of 14 days, and media from replicates of the same sample and treatment were pooled (Fig. 1b). HFA tissue was cultured in MEMα media supplemented with 1× MEM non-essential amino acids, 2 mM sodium pyruvate, 2 mM L-glutamine, 1× Insulin, Transferrin and Selenium (ITS) supplement, 1× Penicillin/Streptomycin, and 10% Fetal Bovine Serum (FBS). All cell media and supplements were from Gibco (Naerum, Denmark), except ITS (Sigma-Aldrich, Broendby, Denmark). During the final 6 h, all tissue fragments were cultured in media containing BrdU labeling reagent (Life Technologies, Naerum, Denmark) to identify proliferation at the end of the culture period as previously described [17].

### *Ex vivo* culture treatments

The effects of the following selected steroid enzyme inhibitors were examined alone (basal conditions) or with ACTH (1 nM, stimulated conditions): Abiraterone acetate (1 μM), Osilodrostat (1 μM), and Efavirenz (10 μM). Treatment concentrations were selected based on pilot experiments testing doses of 1 and 10 μM which have previously been reported to inhibit steroidogenesis in vitro [9, 19–21] and the lowest dose showing an effect on steroidogenesis was chosen. Additionally, dose-response effect was examined for Abiraterone acetate (1 and 10 μM) and Osilodrostat (1 and 10 μM) under basal conditions. Two adrenals from the same fetus were used in experimental setup with one adrenal used to examine treatment effects under basal conditions through allocation of the tissue into a vehicle (dimethyl sulfoxide (DMSO), 0.1%) and an inhibitor treatment group. The other adrenal was used to examine treatment effects under stimulated conditions through allocation of the tissue into an ACTH-stimulated (DMSO+ACTH) and a stimulated + inhibitor treatment group. ACTH, Abiraterone acetat, Efavirenz, and DMSO were purchased from Sigma-Aldrich (Broendby, Denmark) and Osilodrostat from MedChemExpress (Sollentuna, Sweden).

### LC-MS/MS analysis of culture media

Steroid hormone levels were measured in culture media. The culture media was collected every 48 h and pooled throughout the 14 days culture period for all tissue fragments originating from the same adrenal sample and treatment group. Steroid hormone levels (nM concentrations) were measured using a method established to quantify steroid metabolites in serum by isotope-dilution TurboFlow-LC-MS/MS, as previously described [22]. The method was modified for measurement in culture media [17]. This clinically validated analysis package includes the classic androgens: testosterone, androstenedione, and dehydroepiandrosterone-sulfate (DHEAS); glucocorticoids: cortisone and cortisol; and the steroidogenic intermediates: 11-deoxycortisol, 17-hydroxyprogesterone, progesterone, and corticosterone (classified as an intermediate downstream the mineralocorticoid pathway despite being a potent glucocorticoid in vivo [23]. All measured steroids, except estrone sulfate, are reported in this study. In brief, samples were analyzed in five batches: one during the winter of 2019, three during the summer/autumn of 2020, and one during the spring of 2021. For all batches, two blanks (water), two un-spiked media controls, two spiked media controls with a mixture of native steroid standards in low concentrations, and two spiked media controls with the native steroid standards in high concentrations were used as method controls, while standards prepared in media were used for calibration curves. For several samples, the concentrations of DHEAS, cortisol, and cortisone were out of the standard measurement range and were therefore diluted 1:10 with media and analyzed in a repeated batch. For all analytical batches included in this study, the relative standard deviation (RSD) was < 10% for all steroids in culture media controls spiked in low level, except for DHEAS (< 17%), while RSD was < 4.2% for all steroids in the controls spiked in high level. Standard ranges and limit of detection for each of the measured steroids are listed in Additional file 1: Table S1.

### Immunohistochemistry and immunofluorescence

Immediately after the *ex vivo* culture period, HFA tissue fragments were fixed in formalin followed by dehydration and paraffin embedding according to standard procedures. Serial sections (4 μm) were dewaxed and rehydrated prior to immunohistochemistry (IHC) or immunofluorescence (IF) protocols. In both protocols, tissue sections were subjected to heat-induced antigen retrieval in a pressure cooker, peroxidase block in 1% and 3% (v/v) H_2_O_2_ in MeOH for 30 min for the IHC and IF protocols, respectively, followed by incubation in horse serum (20% v/v) with PBS/BSA (5% w/v) (HS) ImmPRESS (Vector Laboratories, Burlingame, California). Tissue sections were washed in Tris-buffered saline between protocol steps and all incubations were carried out in a humidity box.

IHC was conducted as previously described for formalin samples [3]. In brief, after incubation with HS, tissue sections were incubated with primary antibodies diluted in HS overnight at 4 °C, followed by 1 h at room temperature. Finally, sections were incubated for 30 min with the appropriate ImmPRESS HPR (peroxidase, Vector Laboratories, Burlingame, California) secondary antibody. Visualization was performed using ImmPACT AEC peroxidase substrate (Vector Laboratories, Burlingame, California). Sections were counterstained with Mayer’s hematoxylin before mounting with Aquatex (Merck, Darmstadt, Germany). Sections were initially evaluated on a Nikon Microphot-FXA microscope and then by scanning slides on a NanoZoomer 2.0 HT (Hamamatsu Photonics, Herrsching am Ammersee, Germany).

Triple IF was done as previously described for formalin samples [24]. In brief, tissue sections were incubated overnight at 4 °C with primary antibody each diluted in HS. Subsequently sections were incubated with peroxidase-conjugated secondary antibody diluted in HS for 30 min at room temperature, followed by incubation with Tyr-Cy3 according to manufacturer’s instructions. Prior to addition of the 2nd and 3rd primary antibody, respectively, sections were again subjected to heat-induced antigen retrieval buffer in a pressure cooker followed by blocking in HS. Finally, sections were counterstained with DAPI diluted in PBS for 10 min before mounting with Permafluor (Thermo Scientific, UK). Fluorescent images were captured using an Olympus BX61 microscope (Olympus). The first primary antibody applied is as follows: CYP11B2, incubated with peroxidase-conjugated chicken ant-rabbit secondary antibody and Perkin Elmer-TSA- Plus Cyanine3. The second primary antibody applied is as follows: CYP17A1, incubated with peroxidase-conjugated chicken anti-rabbit secondary antibody and Perkin Elmer-TSA-Plus Fluorescein System. The third primary antibody applied is as follows: CYP21A2, incubated with peroxidase-conjugated chicken anti-goat secondary antibody and Perkin Elmer-TSA-Plus Cyanine5 System. All secondary peroxidase-conjugated chicken antibodies were from Santa Cruz Biotechnology (Heidelberg, Germany), and TSA-Plus HPR labeled reagents were from Perkin Elmer Life Sciences (Boston, MA, USA).

Primary antibody dilutions and retrieval buffers for IHC and IF are listed in Table 1. Negative controls were included and processed with the primary antibody replaced by the dilution buffer alone, none of which showed staining.

**Table 1:** Antibodies, dilutions, and retrieval buffers used

Antibody	Dilution	Retrieval buffer	Species	Supplier	Number
BrdU	1:100	CIT	Mouse	Dako	M0744
cPARP	1:500	CIT	Rabbit	Cell Signaling	5625
CYP17A1	1:1,500	CIT	Rabbit	Abcam	Ab134910
CYP11B2*	1:200	TEG	Rabbit	Sigma	HPA049171
CYP21A2	1:15,000	TEG	Goat	Santa Cruz	Sc-48466

TEG buffer: 10 mM Tris, 0.5 mM EGTA, pH 9.0; citrate (CIT) buffer: 10 mM, pH 6.0. *The antigen sequence is 96% identical with CYP11B1

### Statistics

Treatment effects on steroid hormone levels under basal and ACTH-stimulated conditions were investigated as ratios relative to the mean of vehicle controls. To meet normality of residuals and homogeneity of variance, ratios were transformed by the natural logarithm (ln). Treatment effects were always compared with vehicle controls (DMSO) or stimulated controls (ACTH+DMSO) from the same fetus. Effects of simultaneous inhibitor and ACTH treatment were always compared with effects of ACTH-stimulation alone from the same fetus. Therefore, effects of inhibitors under basal and ACTH-stimulated conditions were analyzed based on the ln-transformed data using one-way ANOVA for repeated measures followed by Tukey’s multiple comparisons test. Because dose-response effects of low and high inhibitor doses were not always examined in the same fetus, dose-response effects were analyzed based on the ln-transformed data of ratios relative to the internal vehicle controls using one-way ANOVA followed by Tukey’s multiple comparisons test. No outliers were excluded from the dataset. All tests were two-tailed and *p* < 0.05 were considered statistically significant. Treatment effects were back-transformed to obtain the fold change, which was graphically illustrated with data represented as geometric mean with 95% CI. Each sample measurement represents the mean value of all tissue fragments originating from the same sample and treatment group (considered as technical replicates). Statistical analysis and graphical illustrations were performed using GraphPad Prism Software.

## Results

### Tissue viability and steroid enzyme expression is maintained following treatment with steroidogenic inhibitors

**Fig. 2:**
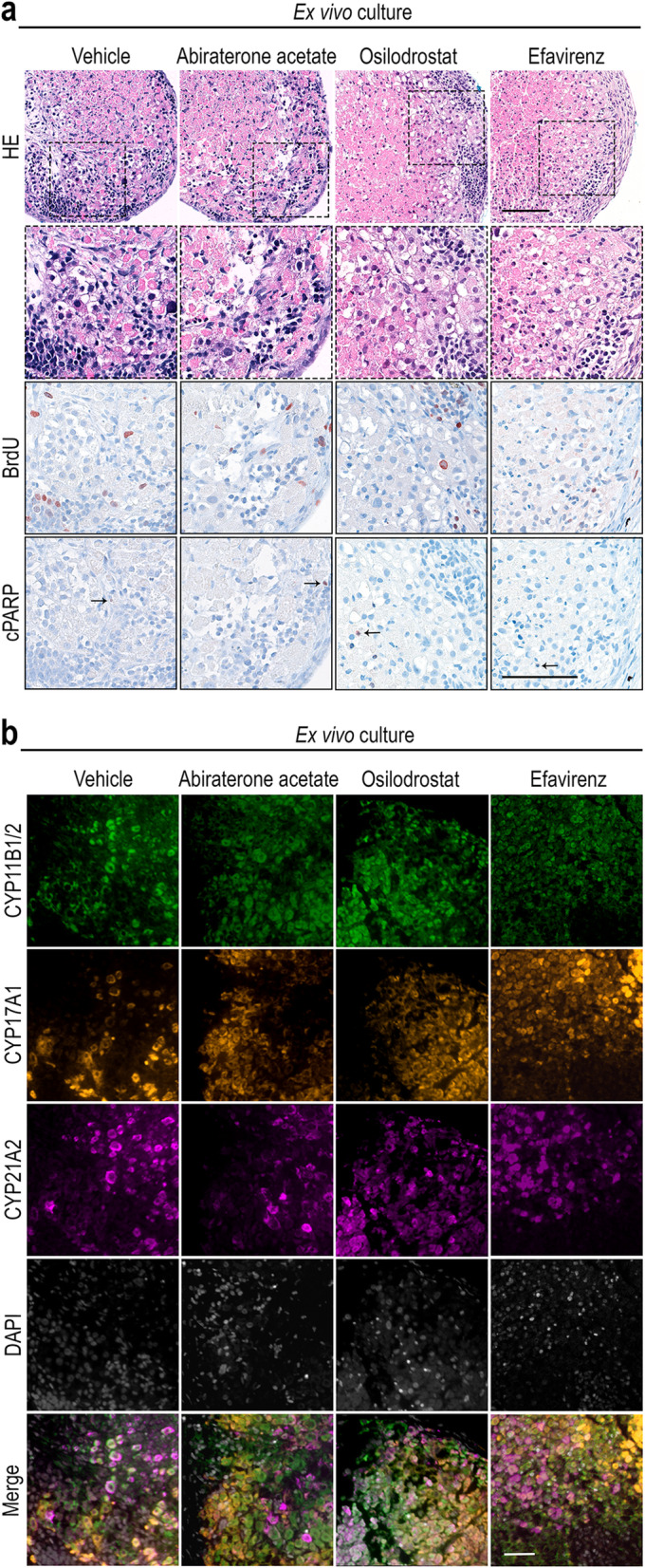
Effects of the selected steroidogenic inhibitors on cell viability and expression of steroid enzyme expression. **a** Morphology and expression of BrdU^+^ (proliferation marker) and cPARP^+^ cells (indicated by arrows, apoptosis marker) investigated on serial sections of fetal adrenal tissue cultured *ex vivo* with Abiraterone acetate (1 μM), Osilodrostat (1 μM), or Efavirenz (10 μM) for 14 days (**a**). Counterstaining with Mayer’s hematoxylin. **b** Triple immunofluorescence with CYP11B1/2 (green), CYP17A1 (orange), CYP21A2 (purple), and DAPI (gray, DNA marker). Age of fetal samples shown (at the start of experiment): vehicle control GW 11 + 3; Abiraterone acetate GW 11 + 3; Osilodrostat GW 11 + 2; Efavirenz GW 9. Scale bars corresponds to 100 μm

Examination of the cultured tissue fragments was used to determine whether the investigated concentrations of the selected inhibitors affected HFA tissue morphology and viability. Overall, no apparent changes in tissue morphology were observed in the inhibitor-treated samples compared with vehicle controls (Fig. 2a). Likewise, in cultured tissue fragments treated with Abiraterone acetate, Osilodrostat, or Efavirenz proliferating cells (BrdU^+^) were present, while no or few apoptotic (cPARP^+^) cells were observed which was similar to vehicle controls. Treatment with a 10-fold higher dose of Abiraterone acetate and Osilodrostat also did not affect proliferation and apoptosis (based on BrdU^+^ and cPARP^+^ cells) compared to vehicle controls (data not shown). This together suggests that the effects of the selected inhibitors on adrenal steroidogenesis were not due to cytotoxicity. The steroidogenic enzymes expected to be inhibited by the selected inhibitors were all expressed in the *ex vivo* cultured tissue samples after the 14 days *ex vivo* culture period (Fig. 2b). Since no apparent difference in expression pattern or level compared to vehicle controls was observed, effects of the examined inhibitors are suggested to be antagonistic rather than affecting the expression levels of the steroidogenic enzymes.

### Effects of Abiraterone acetate treatment on human fetal adrenal steroidogenesis

**Fig. 3:**
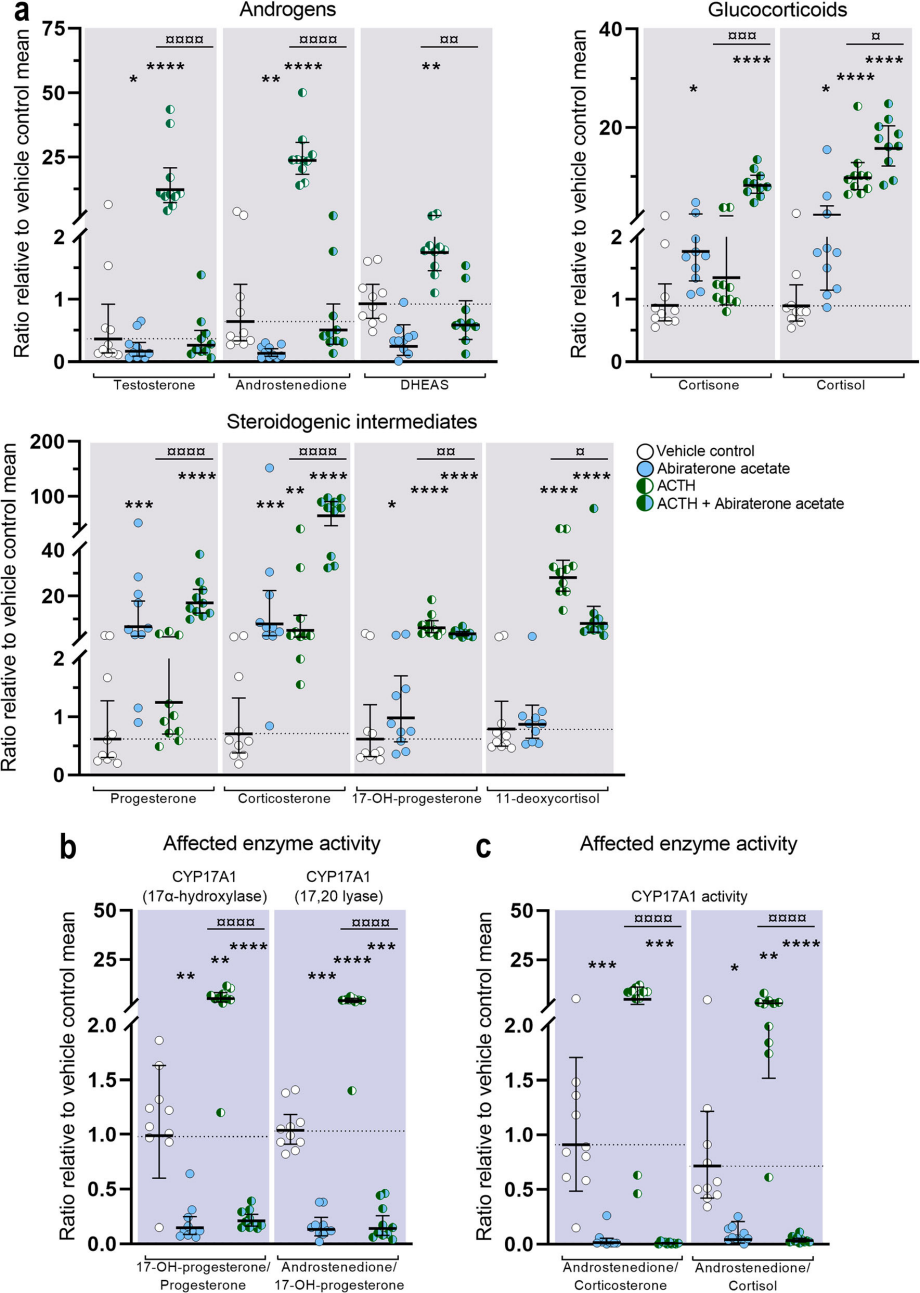
Effects of Abiraterone acetate inhibition on classic steroidogenesis in human fetal adrenals. Effects of Abiraterone acetate (1 μM) on HFA tissue cultured *ex vivo* for 14 days under basal and ACTH-stimulated conditions. **a** Quantification of androgens, glucocorticoids, and steroidogenic intermediate concentrations presented as a ratio relative to the mean of vehicle controls (indicated by dotted lines). **b** Affected enzyme activity reflected by CYP17A1 product/substrate ratios of measured steroids relative to the mean of vehicle controls. **c** Affected CYP17A1 enzyme activity reflected by ratios representing changes in androstenedione/corticosteroids downstream of the mineralocorticoid (corticosterone) and glucocorticoid (cortisol) pathways. Media was pooled from 1 to 13 tissue fragments per treatment depending on the initial size of half of the adrenal gland. Values represent geometric mean with 95% CI, *n* = 10 (fetuses). Significant difference compared with vehicle controls from the same fetus were based on ln-transformed data using repeated measures one-way ANOVA followed by Tukey’s multiple comparisons test. (*) indicate differences compared with vehicle controls, (¤) indicate differences compared with ACTH treatment. *^/¤^*p* < 0.05, **^/¤¤^*p* < 0.01, ***^/¤¤¤^*p* < 0.001, ****^/¤¤¤¤^*P* < 0.0001. DHEAS, dehydroepiandrosterone-sulfate

Treatment with Abiraterone acetate (1 μM) affected the secretion of all investigated steroids. Specifically, Abiraterone acetate caused decreased androgen levels, a small rise in glucocorticoid levels, and a considerable increase in the level of some steroidogenic intermediates under basal conditions (Fig. 3a). The inhibitory effect on HFA androgen biosynthesis was evident from reduced levels of testosterone (2.2-fold decrease, *p* < 0.05) and androstenedione (4.9-fold decrease, *p* < 0.01) and a tendency towards decreased DHEAS levels although this was not statistically significant. Abiraterone acetate treatment increased the levels of most measured glucocorticoids, including cortisone (2.0-fold, *p* < 0.05) and cortisol (2.4-fold, *p* < 0.05), and further resulted in a substantial increase in the secretion of the steroidogenic intermediates progesterone (11-fold, *p* < 0.001), corticosterone (11-fold, *p* < 0.001), and 17-hydroxyprogesterone (1.6-fold, *p* < 0.05). Treatment with Abiraterone acetate (1 μM) under ACTH-stimulated conditions affected the secretion of all measured steroids compared with effects of ACTH-stimulation alone, including DHEAS and 11-deoxycortisol that were not altered after Abiraterone acetate treatment under basal conditions (Fig. 3a). In particular, the inhibitory effect on adrenal androgen biosynthesis was more pronounced under ACTH-stimulated conditions with reduced biosynthesis of testosterone (47-fold decrease, *p* < 0.0001), androstenedione (47-fold decrease, *p* < 0.0001), and DHEAS (3.0-fold decrease, *p* < 0.01) when compared with ACTH-stimulation alone. Also, the effects of Abiraterone acetate under stimulated conditions caused elevated levels of cortisone (6.1-fold, *p* < 0.001) and cortisol (1.6-fold, *p* < 0.05), while the glucocorticoid intermediates 11-deoxycortisol (3.5-fold decrease, *p* < 0.05) and 17-hydroxyprogesterone (1.7-fold decrease, *p* < 0.01) levels were reduced under ACTH-stimulated conditions. Finally, the levels of progesterone (14-fold, *p* < 0.0001) and corticosterone (13-fold, *p* < 0.0001) increased following Abiraterone acetate treatment under stimulated conditions.

The inhibitory effect of Abiraterone acetate under basal and ACTH-stimulated conditions was further evident from the CYP17A1 product/substrate ratios (Fig. 3b). Thus, the 17α-hydroxylase product/substrate ratio (17-hydroxyprogesterone/progesterone) decreased 6.8-fold (*p* < 0.01) under basal conditions and 24-fold (*p* < 0.0001) under ACTH-stimulated conditions. Accordingly, the 17,20-lyase product/substrate ratio (androstenedione/17-hydroxyprogesterone) decreased 7.8-fold (*p* < 0.001) under basal conditions and 27-fold (*p* < 0.0001) under ACTH-stimulated conditions. A potent androgen inhibition was also evident from the ratios reflecting the combined CYP17A1 activity after Abiraterone acetate treatment (Fig. 3c). The androstenedione/corticosterone ratio decreased 55-fold (*p* < 0.001) under basal conditions and 608-fold (*p* < 0.001) under ACTH-stimulated conditions and the androstenedione/cortisol ratio decreased 12-fold (*p* < 0.0001) under basal conditions and 76-fold (*p* < 0.0001) under ACTH-stimulated conditions.

The observed increase in HFA glucocorticoid levels upon treatment with Abiraterone acetate (1 μM) led us to speculate whether this could be the result of incomplete CYP17A1 inhibition. Therefore, the effects of a 10-fold higher concentration of Abiraterone acetate were examined under basal conditions in HFA tissue cultured *ex vivo* for 14 days (Additional file 2: Figure S1). Treatment with Abiraterone acetate (10 μM) reduced the levels of testosterone (3.4-fold decrease, *p* < 0.001), androstenedione (30-fold decrease, *p* < 0.0001), and DHEAS (24-fold decrease, *p* < 0.0001) in accordance with the observations from treatment with the lower concentration of Abiraterone acetate (1 μM). However, the 10 μM dose further decreased androstenedione levels 5.9-fold (*p* < 0.0001) and DHEAS levels 10-fold (*p* < 0.0001) compared with treatment effects of the 1 μM dose. Interestingly, the 10-fold higher concentration of Abiraterone acetate shifted the effect from an increase (at 1 μM) to a decrease (at 10 μM) in the secretion of glucocorticoids, including reduced levels of cortisone (3.7-fold decrease, *p* < 0.0001), cortisol (2.6-fold decrease, *p* < 0.01), and the intermediate 11-deoxycortisol (8.0-fold decrease, *p* < 0.0001), compared with vehicle control. Thus, the increased Abiraterone acetate concentration resulted in a 7.2-fold decrease in cortisone levels (*p* < 0.0001), 6.2-fold decrease in cortisol levels (*p* < 0.0001), and an 11-fold decrease in 11-deoxycortisol levels (*p* < .0001) compared with treatment effects of 1 μM Abiraterone acetate. Abiraterone acetate treatment (10 μM) also increased the levels of the steroidogenic intermediate progesterone (68-fold, *p* < 0.0001) and corticosterone (15-fold, *p* < 0.0001) causing an additional 3.2-fold increase (*p* < 0.0001) in progesterone levels compared with treatment effects following the 1 μM dose.

### Effects of Osilodrostat treatment on human fetal adrenal steroidogenesis

**Fig. 4:**
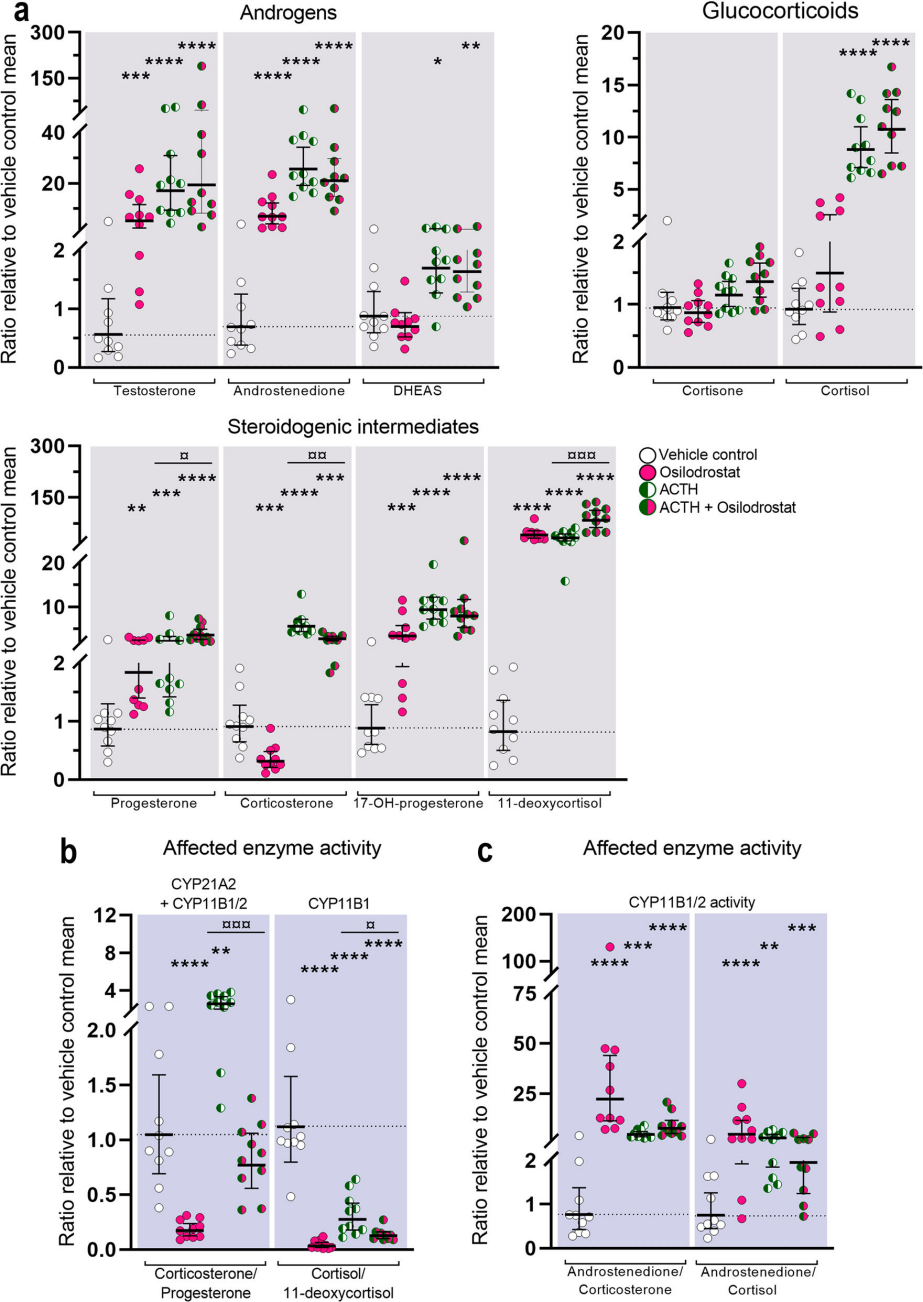
Effects of Osilodrostat inhibition on classic steroidogenesis in human fetal adrenals. Effects of Osilodrostat (1 μM) on HFA tissue cultured *ex vivo* for 14 days under basal and ACTH-stimulated conditions. **a** Quantification of androgens, glucocorticoids, and steroidogenic intermediate concentrations presented as a ratio relative to the mean of vehicle controls (indicated by dotted lines). **b** Affected enzyme activity reflected by CYP11B1/2 product/substrate ratios of measured steroids relative to the mean of vehicle controls. **c** Affected CYP11B1/2 enzyme activity reflected by ratios representing changes in androstenedione/corticosteroids downstream of the mineralocorticoid (corticosterone) and glucocorticoid (cortisol) pathways. Media was pooled from 1 to 13 tissue fragments per treatment depending on the initial size of half of the adrenal gland. Values represent geometric mean with 95% CI, *n* = 10 (fetuses). Significant difference compared with vehicle controls from the same fetus were based on ln-transformed data using repeated measures one-way ANOVA followed by Tukey’s multiple comparisons test. (*) indicate differences compared with vehicle controls, (¤) indicate differences compared with ACTH treatment. *^/¤^*p* < 0.05, **^/¤¤^*p* < 0.01, ***^/¤¤¤^*p* < 0.001, ****^/¤¤¤¤^*P* < 0.0001. DHEAS, dehydroepiandrosterone-sulfate

Treatment with Osilodrostat (1 μM) altered the secretion of the investigated steroidogenic intermediates and androgens under basal conditions in *ex vivo* cultured HFA tissue (Fig. 4a). Thus, treatment with Osilodrostat affected HFA androgen secretion causing an increase in testosterone (9.5-fold, *p* < 0.001) and androstenedione (10.3-fold, *p* < 0.0001), while DHEAS levels were unaffected. Although no effects were detected on the secretion of cortisone and cortisol, Osilodrostat treatment did result in increased levels of glucocorticoid intermediates upstream of CYP11B1 activity. This increase in the levels of intermediates included 17-hydroxyprogesterone (3.8-fold, *p* < 0.001) and the 11β-hydroxylase substrate 11-deoxycortisol (50-fold, *p* < 0.0001). Additionally, treatment with Osilodrostat reduced corticosterone levels (2.9-fold decrease, *p* < 0.001) and increased progesterone levels (2.1-fold, *p* < 0.01) under basal conditions. ACTH-stimulation prevented the treatment-induced increase in adrenal androgens and glucocorticoid intermediates levels that was observed following treatment with Osilodrostat (1 μM) under basal conditions (Fig. 4a). Thus, no effects of Osilodrostat treatment were observed on adrenal androgen levels under stimulated conditions compared with ACTH-stimulation alone, while the increase in 11β-hydroxylase substrate 11-deoxycortisol (2.5-fold, *p*< 0.001) was less pronounced than the response under basal conditions. Following ACTH-stimulation, only effects on corticosterone (2.1-fold decrease, *p* < 0.01) and progesterone (1.6-fold increase, *p* < 0.05) levels were of similar level as the effects of Osilodrostat treatment under basal conditions.

The effect of Osilodrostat treatment on CYP11B1/2 product/substrate ratios showed effective inhibition under both basal and ACTH-stimulated conditions (Fig. 4b). The corticosterone/progesterone ratio decreased 6.1-fold (*p* < 0.0001) under basal and 3.4-fold (*p*< 0.001) under ACTH-stimulated conditions. Additionally, the cortisol/11-deoxycortisol ratio decreased 31-fold (*p* < 0.0001) under basal conditions and 2.1-fold (*p* < 0.05) under ACTH-stimulated conditions. The effect on steroidogenic intermediates and glucocorticoid levels under basal conditions was also clear from the ratios reflecting the combined CYP11B1/2 activity (Fig. 4c) with increased androstenedione/corticosterone (29-fold, *p* < 0.0001) and androstenedione/cortisol ratios (6.3-fold *p* < 0.001) under basal conditions, while no effects on these ratios were observed under ACTH-stimulated conditions (Fig. 4c).

Treatment with Osilodrostat (1 μM) did not lead to a decrease in secretion of cortisol in *ex vivo* cultured HFA tissues as has previously been reported in patient studies [25]. Since Osilodrostat is known to be a more potent inhibitor of aldosterone synthase activity than 11β-hydroxylase activity [26], we speculated whether the effect of Osilodrostat on glucocorticoid secretion could be dose-dependent. Therefore, the effects of a 10-fold higher concentration of Osilodrostat were examined under basal conditions in HFA tissue cultured *ex vivo* for 14 days (Additional file 3: Figure S2). Treatment with Osilodrostat resulted in a dose-dependent inhibition of HFA glucocorticoid secretion. Interestingly, the higher concentration of Osilodrostat (10 μM) caused a reduction in the levels of both cortisone (1.5-fold decrease, *p* < 0.05) and cortisol (3.6-fold decrease, *p* < 0.01) compared with vehicle controls, and a 5.4-fold decrease in cortisol levels (*p* < 0.0001) compared with treatment effects of 1 μM Osilodrostat. Treatment with Osilodrostat (10 μM) also increased the levels of steroidogenic intermediates upstream of 11β-hydroxylase activity (11-deoxycortisol 31-fold, *p* < 0.0001; and 17-hydroxyprogesterone 2.7-fold *p* < 0.0001) in accordance with the observations from treatment with the lower concentration of Osilodrostat (1 μM). The increase in progesterone (5.3-fold, *p* < 0.0001) and reduction in corticosterone levels (25-fold decrease, *p* < 0.0001) compared with vehicle controls were 2.5-fold higher for progesterone (*p* < 0.001) and 9.1-fold lower for corticosterone (*p* < 0.0001) compared with treatment effects of 1 μM Osilodrostat. Treatment with Osilodrostat (10 μM) increased the levels of testosterone (3.4-fold, *p* < 0.01), androstenedione (3.8-fold, *p* < 0.0001), and reduced the levels of DHEAS (2.2-fold decrease, *p* < 0.01) compared with vehicle controls. This corresponded to a 2.6-fold decrease of testosterone (*p* < 0.05) and 2.3-fold decrease in androstenedione (*p* < 0.001) levels compared with treatment effects of 1 μM Osilodrostat.

### Effects of Efavirenz treatment on human fetal adrenal steroidogenesis

**Fig. 5:**
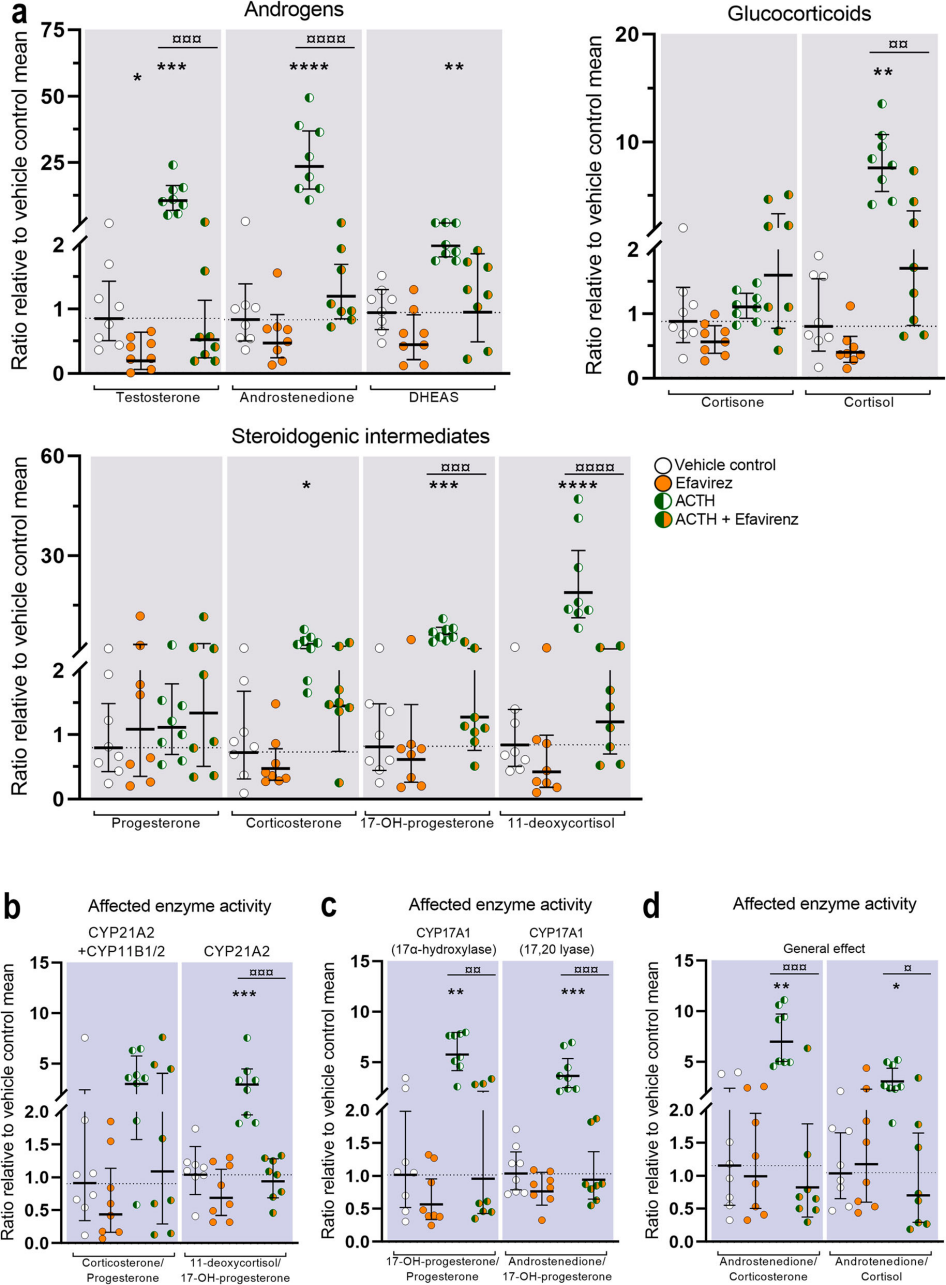
Effects of Efavirenz inhibition on classic steroidogenesis in human fetal adrenals. Effects of Efavirenz (10 μM) on HFA tissue cultured *ex vivo* for 14 days under basal and ACTH-stimulated conditions. **a** Quantification of androgens, glucocorticoids, and steroidogenic intermediate concentrations presented as a ratio relative to the mean of vehicle controls (indicated by dotted lines). Affected enzyme activity reflected by CYP21A2 (**b**) and CYP17A1 (**c**) product/substrate ratios of measured steroids relative to the mean of vehicle controls. **d** General treatment effect reflected by ratios representing changes in androstenedione/corticosteroids downstream the mineralocorticoid (corticosterone) and glucocorticoid (cortisol) pathways. Media was pooled from 1 to 13 tissue fragments per treatment depending on the initial size of half of the adrenal gland. Values represent geometric mean with 95% CI, *n* = 8 (fetuses). Significant difference compared with vehicle controls from the same fetus were based on ln-transformed data using repeated measures one-way ANOVA followed by Tukey’s multiple comparisons test. (*) indicate differences compared with vehicle controls, (¤) indicate differences compared with ACTH treatment. *^/¤^*p* < 0.05, **^/¤¤^*p* < 0.01, ***^/¤¤¤^*p* < 0.001, ****^/¤¤¤¤^*P* < 0.0001. DHEAS, dehydroepiandrosterone-sulfate

Treatment with Efavirenz (10 μM) in *ex vivo* cultured HFA tissue only affected the secretion of testosterone (4.3-fold decrease, *p* < 0.05) under basal conditions, with no statistically significant effects observed on glucocorticoid and steroidogenic intermediate levels (Fig. 5a). However, under ACTH-stimulated conditions, treatment with Efavirenz inhibited the biosynthesis of both adrenal androgens, glucocorticoids and steroidogenic intermediates when compared with ACTH-stimulation alone (Fig. 5a). Under stimulated conditions, Efavirenz reduced the levels of testosterone (20-fold decrease, *p* < 0.001) and androstenedione (20-fold decrease, *p* < 0.0001) as well as the levels of cortisol (4.4-fold decrease, *p* < 0.01), 11-deoxycortisol (16-fold decrease, *p* < 0.0001) and 17-hydroxyprogesterone (5.0-fold decrease, *p* < 0.001).

The unspecific inhibitory effect of Efavirenz observed under ACTH-stimulated conditions was further evident from the product/substrate ratios reflecting the activity of CYP21A2 and CYP17A1. The corticosterone/progesterone ratio reflecting CYP21A2 and CYP11B1/2 activity was unaffected by Efavirenz under both basal and stimulated conditions (Fig. 5b), while the 11-deoxycortisol/17-hydroxyprogesterone ratio reflecting decreased CYP21A2 activity (3.1-fold *p* < 0.001) under ACTH-stimulated conditions (Fig. 5b). Also, CYP17A1 product/substrate ratio was unaffected by Efavirenz under basal conditions (Fig. 5c), while under ACTH-stimulated conditions both the 17-hydroxyprogesterone/progesterone (6.0-fold, *p* < 0.01) and androstenedione/17-hydroxyprogesterone (3.9-fold, *p*< 0.001) ratios were decreased (Fig. 5c). The overall inhibitory effect of Efavirenz on adrenal steroidogenic enzymes was further evident from the 8.5-fold decrease (*p* < 0.001) in the androstenedione/corticosterone ratio and a 4.4-fold decrease (*p* < 0.05) in the androstenedione/cortisol ratio under stimulated conditions (Fig. 5d). Thus, treatment with Efavirenz under stimulated conditions appears to inhibit androgen rather than corticosteroid biosynthesis suggesting unspecific inhibition of several steroidogenic enzymes rather than specific CYP21A2 inhibition.

## Discussion

This study used an established HFA *ex vivo* culture model [17] to investigate the effects of three therapeutic drugs selected to inhibit different steps in adrenal steroidogenesis. Each inhibitor was examined under both basal and ACTH-stimulated conditions to determine how this impacted the inhibitor-mediated effects on adrenal steroidogenesis. Based on the steroid hormone profiles determined in this study, the well-described inhibitors Abiraterone acetate and Osilodrostat showed specific inhibition of CYP17A1 and CYP11B1/2, respectively, while treatment with Efavirenz resulted in unspecific inhibition of adrenal steroidogenesis instead of the expected CYP21A2 inhibition. Importantly, all therapeutic drugs showed different responses under basal and stimulated conditions. None of the treatment concentrations used to examine inhibition of steroidogenesis showed any apparent negative effects on tissue viability. Additionally, the protein expression of enzymes expected to be inhibited by the selected drugs was examined and appeared to be similar in expression level pattern compared to controls. Therefore, the investigated drugs are most likely inhibiting steroidogenic enzyme activity rather than reducing enzyme expression in the HFA tissue, which is in accordance with the inhibitory mechanisms described for Abiraterone acetate (an irreversible inhibitor [27]) and Osilodrostat (a reversible competitive inhibitor [28]).

Abiraterone acetate is a highly selective potent inhibitor of 17α-hydroxylase and 17,20-lyase activities [29]. This CYP17A1 inhibitor is used to treat androgen-dependent diseases like castration-resistant prostate cancer [30] and has been used to efficiently normalize excess circulating androgen levels in CAH patients [31]. Accordingly, Abiraterone acetate efficiently inhibited 17α-hydroxylase and 17,20-lyase activities in the *ex vivo* cultured HFA tissue based on the observed steroid hormone profiles and in particular from the decreased CYP17A1 product/substrate ratios. Thus, treatment with Abiraterone acetate caused decreased androgen secretion and increased levels steroidogenic intermediates, which is in accordance with previously described effects in castration-resistant prostate cancer patients [29]. The potent androgen inhibition of Abiraterone acetate was also evident in the decreased ratios of androstenedione/corticosterone and androstenedione/cortisol, suggesting a shift in the adrenal biosynthesis towards corticosteroid biosynthesis.

The effect of Abiraterone acetate on HFA glucocorticoid biosynthesis was dose-dependent with a shift from increased to decreased glucocorticoid levels observed when increasing the Abiraterone acetate dose 10-fold (from 1 μM to 10 μM). This may be the result of a shift from an accumulation of stalled steroid precursors before the 17,20-lyase activity which could mediate the conversion into glucocorticoids following treatment with the low dose, to an accumulation of steroid precursors which are stalled before the initial 17α-hydroxylase activity of CYP17A1 following treatment with the high dose.

Interestingly, Abiraterone acetate treatment resulted in more potent inhibition of CYP17A1 under ACTH-stimulated conditions in the *ex vivo* cultured HFA tissue. Thus, our results suggesting that the effect of Abiraterone acetate-mediated inhibition of CYP17A1 enzyme activity is altered by ACTH-stimulation indicate that models used to test emerging steroidogenic inhibitors as well as adrenal disease models should consider the impact of endogenous ACTH levels when assessing the effects of enzyme activity.

Abiraterone acetate has previously been suggested to inhibit CYP17A1 and CYP21A2 in studies with primary canine adrenocortical cells and human NCI-H295R cells [19, 20]. However, the observed increase in corticosterone levels following treatment with Abiraterone acetate in the ex vivo cultured HFA tissue does not support inhibition of CYP21A2 since the biosynthesis of corticosterone is dependent on initial 21-hydroxylation of progesterone. This difference could be the result of species-specific differences in adrenal steroidogenesis despite canines also requiring CYP17A1 to complete glucocorticoid biosynthesis. The discrepancy with results from NCI-H295R cells may be due to the modest response of these cells to ACTH-stimulation, which limits the usefulness of this cell line in mimicking an active HPA axis [32] and emphasize the importance of a steroidogenic human model that responds to ACTH-stimulation when examining the inhibitory effects of drugs targeting adrenal steroidogenic enzymes.

Osilodrostat has been reported to inhibit the aldosterone synthase activity of CYP11B2 and at higher doses also the 11β-hydroxylase activity of CYP11B1/2 [26]. In *ex vivo* cultured HFA tissue, Osilodrostat treatment increased the levels of androgens and glucocorticoid precursors upstream of 11β-hydroxylase activities, while corticosterone, which is downstream the 11β-hydroxylase activity of CYP11B1/2, was decreased under basal conditions. These findings are in accordance with accumulation of precursors including 11-deoxycortisol and increased adrenal androgen levels in Cushing’s disease patients treated with Osilodrostat [25, 26]. Despite potent inhibition of CYP11B1/2 product/substrate ratios which showed reduced 11β-hydroxylase activity, the overall decrease in secretion of glucocorticoids downstream of CYP11B1 activities were only significant after treatment with the high dose (10 μM) of Osilodrostat. This does-dependent response observed in the *ex vivo* cultured HFA tissue is in accordance with the previous reported dose-dependent inhibition of CYP11B2 and CYP11B1 [26]. This suggests that the low dose of Osilodrostat (1 μM) used in these experiments was not sufficient to inhibit CYP11B1 completely but high enough to inhibit CYP11B2 enzyme activity resulting in the reduced biosynthesis of corticosterone and possibly aldosterone (although not measured in this study). Accordingly, the dose of Osilodrostat needed to normalize urinary free cortisol levels in patients and to decrease glucocorticoid secretion in primary adrenal patient cultures have previously been shown to be highly variable [19, 25]. Furthermore, the decrease in DHEAS and the less pronounced increase in both androstenedione and testosterone following treatment with 10 μM Osilodrostat suggest that the high dose not only inhibit CYP11B1 but may also affect the steroidogenic activity of CYP17A1 as described previously [21].

In contrast to the observations from Abiraterone acetate treatment, ACTH-stimulation reduced the inhibitory effect of Osilodrostat on CYP11B1 activity thereby affecting the glucocorticoid pathway, while the inhibitory effect on CYP11B2 under basal conditions was comparable to the effect under ACTH-stimulated conditions. This observation is in accordance with in vitro studies of cortisol secretion in the HCA15 cell line showing increased IC_50_ values of Osilodrostat upon ACTH-stimulation compared with IC_50_ values determined under basal conditions [21]. Thus, our results suggest that treatments with steroidogenic inhibitors may be differently affected by stimulation of the HPA-axis. Hence, the effective dose of Osilodrostat might vary according to etiology, e.g., in primary Cushing patients with suppressed ACTH levels versus pituitary Cushing with chronically high ACTH levels [33].

Efavirenz is a drug typically used as an antiviral HIV treatment, but a recent in vitro study proposed that Efavirenz is also a specific CYP21A2 inhibitor at higher concentrations (10–50 μM) [9]. In the HFA *ex vivo* culture experiments, Efavirenz (10 μM) caused a reduction in the secretion of testosterone under basal conditions. However, under ACTH-stimulated conditions Efavirenz potently reduced the levels of adrenal androgens, cortisol and the glucocorticoid intermediates 11-deoxycortisol and 17-hydroxyprogesterone. The reduced secretion of androgens and the affected product/substrate ratios reflecting 17α-hydroxylase and 17,20-lyase activities indicate inhibition of CYP17A1, while the effects on the other steroid hormone profiles suggest unspecific inhibition causing a decrease in steroids both up- and downstream CYP21A2 activities. The tendency towards reduced corticosterone levels and inhibited 11-deoxycortisol/17-hydroxyprogesterone ratio under ACTH-stimulated conditions from the HFA tissue cultures may support the notion of CYP21A2 inhibition, which was previously reported after Efavirenz treatment in the H295R cell line [32]. In this study, an in vitro assay was also used to determine the inhibitory effect of Efavirenz on recombinant expressed CYP21A2 enzyme substrate conversion of 17-hydroxyprogesterone at doses (50 μM) above the reported effective mean serum concentrations of 1.6–9.1 μM [9]. The H295R cells have a modest response to ACTH [32] and all experiments in the reported in vitro study were therefore conducted under basal conditions. Since the 11-deoxycortisol/17-hydroxyprogesterone ratio was the only reported product/substrate ratio [9], the inhibitory effect on other steroidogenic enzymes in the H295R cell line is unknown. This highlights the importance of analyzing and reporting both steroid hormone profiles and enzyme activity ratios when characterizing potential steroidogenic inhibitors. However, the observed unspecific inhibitory effect on adrenal steroidogenesis in this study does support concerns that Efavirenz may affect adrenal steroidogenesis after pre- and postnatal HIV treatment [9]. Thus, further investigations of the effects of antiviral HIV drugs on adrenal function under basal and stimulated conditions would be highly relevant.

The expression of all steroidogenic enzymes facilitating de novo biosynthesis of steroids from the steroidogenic pathway and the treatment-specific responses reported here and previously [17] suggests that *ex vivo* culture of small HFA tissue fragments is an appropriate model for studying effects of therapeutic drugs on the steroidogenic responses in healthy human adrenal tissue. In accordance with previous characterization [3], no overall age-related differences in steroidogenic activity were found between the 1^st^ trimester samples used in this study, and variance in the data reflects biological variation between HFA samples. However, there are differences between human fetal and adult steroid production most importantly related to the low 3βHSD2 and high SULT2A1 fetal expression that makes it impossible to directly translate results from the HFA *ex vivo* culture model to an in vivo/patient (postnatal) situation. Also, even though HFA secrete C_21_ steroids, the fetal steroid enzyme profile mainly facilitates the production of C_19_ androgens resulting in the abundant secretion of DHEAS [15], which is not representative for the steroid profile of adult adrenal glands. However, the HFA *ex vivo* culture model can be used to examine effects of experimental drugs influencing steroidogenesis which are contraindicated in pregnant women. Importantly, the accordance between the reported steroid hormone profiles from the *ex vivo* cultured HFA tissue and patients treated with Abiraterone acetate and Osilodrostat suggest that it is reasonable to extrapolate the overall treatment response on the different adrenal steroid pathways from the *ex vivo* model to an in vivo situation if cautiously interpreted. Thus, combining the tissue specific biological knowledge from this carefully validated *ex vivo* model with the information of systemic effects of therapy obtained from patient studies might increase the knowledge necessary for optimal treatment monitoring of patients.

## Conclusions

In conclusion, the present study demonstrates specific effects of the well-described inhibitors Abiraterone acetate and Osilodrostat on fetal adrenal steroidogenesis in *ex vivo* culture and further validates the applicability of HFA tissue as a model to examine steroidogenic effects of therapeutic drugs. Importantly, this study demonstrates that the effects of all three selected steroidogenic inhibitors differed under basal and stimulated conditions. Thus, the CYP17A1 inhibition mediated by Abiraterone acetate and the overall inhibitory effects of Efavirenz on steroidogenesis were more potent under ACTH-stimulated conditions, while inhibition of CYP11B1 after treatment with Osilodrostat appeared to be less potent in the ACTH-stimulated condition compared to the basal condition. These treatment-specific differences in inhibitory response under basal and ACTH-stimulated conditions highlights the relevance of combining information about the specific steroidogenic effects obtained from *ex vivo* studies with the systemic responses observed in vivo in patient studies. Thus, obtaining information using both approaches can contribute to an increased biological understanding which may improve treatment strategies and optimize monitoring of patients if subsequently implemented. Hence, the HFA *ex vivo* culture model provides a relevant system to test existing and new treatment options for adrenal steroid hormone-dependent diseases and of drugs with suspected undesired adrenal side effects.

## Supplementary Information

**Additional file 1: Supplementary table.** This file contains an overview of limits of quantification and linear range of detection for the steroid hormones measured by TurboFlow-LC-MS/MS.

**Additional file 2.** Results of the dose-response of Abiraterone acetate treatment. This file shows *ex vivo* cultured HFAs steroid hormone levels following treatment with 1 μM and 10 μM Abiraterone acetate under basal conditions.

**Additional file 3.** Results of the dose-response of Osilodrostat treatment. This file shows *ex vivo* cultured HFAs steroid hormone levels following treatment with 1 μM and 10 μM Osilodrostat under basal conditions.

## Data Availability

All data generated or analyzed during this study are included in this published article and its supplementary information files.

## Abbreviations

ACTH: Adrenocorticotropic hormone
CAH: Congenital adrenal hyperplasia
CIT: Citrate
CS: Cushing syndrome
DHEAS: Dehydroepiandrosterone-sulfate
DMSO: Dimethyl sulfoxide
FBS: Fetal bovine serum
GW: Gestational week
HFA: Human fetal adrenal
HPA: Hypothalamic-pituitary-adrenal
HS: Horse serum
IF: Immunofluorescence
IHC: Immunohistochemistry
ITS: Transferrin and selenium
Ln: Natural logarithm
RSD: Relative standard deviation

## References

[1] Goto M, Piper Hanley K, Marcos J, Wood PJ, Wright S, Postle AD, Cameron IT, Mason JI, Wilson DI, Hanley NA (2006). In humans, early cortisol biosynthesis provides a mechanism to safeguard female sexual development. J Clin Invest..

[2] Johnston ZC, Bellingham M, Filis P, Soffientini U, Hough D, Bhattacharya S (2018). The human fetal adrenal produces cortisol but no detectable aldosterone throughout the second trimester. BMC Medicine..

[3] Melau C, Nielsen JE, Frederiksen H, Kilcoyne K, Perlman S, Lundvall L, Langhoff Thuesen L, Juul Hare K, Andersson AM, Mitchell RT, Juul A, Jørgensen A (2019). Characterization of human adrenal steroidogenesis during fetal development. J Clin Endocrinol Metab..

[4] Savchuk I, Morvan ML, Antignac JP, Gemzell-Danielsson K, le Bizec B, Söder O (2017). Androgenic potential of human fetal adrenals at the end of the first trimester. Endocr Connect..

[5] Cole TJ, Short KL, Hooper SB (2019). The science of steroids. Semin Fetal Neonatal Med..

[6] Bacila IA, Elder C, Krone N (2019). Update on adrenal steroid hormone biosynthesis and clinical implications. Arch Dis Child..

[7] Miller WL, Auchus RJ (2011). The molecular biology, biochemistry, and physiology of human steroidogenesis and its disorders. Endocr Rev..

[8] Merke DP, Auchus RJ (2020). Congenital adrenal hyperplasia due to 21-hydroxylase enzyme deficiency. N Engl J Med..

[9] Malikova J, Zingg T, Fingerhut R, Sluka S, Grössl M, Bernhardt R (2019). HIV drug efavirenz inhibits CYP21A2 activity with possible clinical implications. Horm Res Paediatr..

[10] Reisch N, Taylor AE, Nogueira EF, Asby DJ, Dhir V, Berry A, Krone N, Auchus RJ, Shackleton CHL, Hanley NA, Arlt W (2019). Alternative pathway androgen biosynthesis and human fetal female virilization. PNAS..

[11] Gallo-Payet N (2016). Adrenal and extra-adrenal functions of ACTH. J Mol Endocrinol..

[12] Morsi A, DeFranco D, Witchel SF (2018). The hypothalamic-pituitary-adrenal axis and the fetus. Horm Res Paediatr..

[13] Montenegro YHA, Nascimento DQ, Assis TO, Santos-Lopes SS (2019). The epigenetics of the hypothalamic-pituitary-adrenal axis in fetal development. Ann Hum Genet..

[14] Abbott DH, Zhou R, Bird IM, Dumesic DA, Conley AJ (2008). Fetal programming of adrenal androgen excess: lessons from a nonhuman primate model of polycystic ovary syndrome. Endocr Dev..

[15] Ishimoto H, Jaffe RB (2011). Development and function of the human fetal adrenal cortex: a key component in the feto-placental unit. Endocr Rev..

[16] Mesiano S, Jaffe RB (1997). Developmental and functional biology of the primate fetal adrenal cortex. Endocr Rev..

[17] Melau C, Nielsen JE, Perlman S, Lundvall L, Langhoff Thuesen L, Juul Hare K (2021). Establishment of a novel human fetal adrenal culture model that supports de novo and manipulated steroidogenesis. J Clin Endocrinol Metab.

[18] Evtouchenko L, Studer L, Spencer C, Dreher E, Seiler RW (1996). A mathematical model for the estimation of human embryonic and fetal age. Cell Transplantation..

[19] Sanders K, Wit WL, Mol JA, Kurlbaum M, Kendl S, Kroiss M (2018). Abiraterone acetate for Cushing syndrome: study in a canine primary adrenocortical cell culture model. Endocrinology..

[20] Rijk JCW, Peijnenburg AACM, Blokland MH, Lommen A, Hoogenboom RLAP, Bovee TFH (2012). Screening for modulatory effects on steroidogenesis using the human H295R adrenocortical cell line: a metabolomics approach. Chem Res Toxicol..

[21] Creemers SG, Feelders RA, Jong FH, Franssen GJH, de Rijke YB, Koetsveld PM (2019). Osilodrostat is a potential novel steroidogenesis inhibitor for the treatment of cushing syndrome: an in vitro study. J Clin Endocrinol Metab..

[22] Søeborg T, Frederiksen H, Johannsen TH, Andersson AM, Juul A (2017). Isotope-dilution TurboFlow-LC-MS/MS method for simultaneous quantification of ten steroid metabolites in serum. Clin Chim Acta..

[23] Raubenheimer PJ, Young EA, Andrew R, Seckl JR (2006). The role of corticosterone in human hypothalamic-pituitary-adrenal axis feedback. Clin Endocrinol (Oxf)..

[24] Jørgensen A, Nielsen JE, Perlman S, Lundvall L, Mitchell RT, Juul A, Rajpert-de Meyts E (2015). Ex vivo culture of human fetal gonads: Manipulation of meiosis signalling by retinoic acid treatment disrupts testis development. Hum Reprod..

[25] Bertagna X, Pivonello R, Fleseriu M, Zhang Y, Robinson P, Taylor A, Watson CE, Maldonado M, Hamrahian AH, Boscaro M, Biller BMK (2014). LCI699, a potent 11β-hydroxylase inhibitor, normalizes urinary cortisol in patients with Cushing's disease: results from a multicenter, proof-of-concept study. J Clin Endocrinol Metab..

[26] Duggan S (2020). Osilodrostat: first approval. Drugs..

[27] Reid AMH, Attard G, Barrie E, de Bomo JS (2008). CYP17 inhibition as a hormonal strategy for prostate cancer. Nat Clin Pract Urol..

[28] Ménard J, Rigel DF, Waston C, Jeng AY, Fu F, Beil M (2014). Aldosterone synthase inhibition: cardiorenal protection in animal disease models and translation of hormonal effects to human subjects. J Transl Med..

[29] Vasaitis TS, Bruno RD, Njar VCO (2011). CYP17 inhibitors for prostate cancer therapy. J Stereoid Biochem Mol Biol..

[30] Barnard M, Mostaghel EA, Auchus RJ, Storbeck KH (2020). The role of adrenal derived androgens in castration resistant prostate cancer. J Steroid Biochem Mol Biol..

[31] Auchus RJ, Buschur EO, Chang AY, Hammer GD, Ramm C, Madrigal D, Wang G, Gonzalez M, Xu XS, Smit JW, Jiao J, Yu MK (2014). Abiraterone acetate to lower androgens in women with classic 21-hydroxylase deficiency. J Clin Endocrinol Metab..

[32] Wang T, Rainey WE (2012). Human adrenocortical carcinoma cell lines. Mol Cell Endocrinol..

[33] Braun LT, Reincke M (2020). What is the role of medical therapy in adrenal-dependent Cushing’s syndrome?. Best Pract Res Clin Endocrinol Metab..

