# Supplementary material for: The effects of selected inhibitors on human fetal adrenal steroidogenesis differs under basal and ACTH-stimulated conditions
Source: BMC Med. 2021 Sep 8;19:204. doi: 10.1186/s12916-021-02080-8 (PMC8425147; doi:10.1186/s12916-021-02080-8)
Supplement: Supplementary file 1 — Additional file 1: Supplementary table. This file contains an overview of limits of quantification and linear range of detection for the steroid hormones measured by TurboFlow-LC-MS/MS. [file 12916_2021_2080_MOESM1_ESM.pdf]

**Steroid Hormone**	**LOQ (nM)**	**Linear range (nM)**
Cortisone	0.19	LOQ-200
Cortisol	1.90	LOQ-2000
DHEAS	19.00	LOQ-100,000
Corticosterone	0.10	LOQ-1750
11-deoxycortisol	0.02	LOQ-1750
Δ4-androstenedione	0.04	LOQ-2000
Testosterone	0.01	LOQ-1500
17α-hydroxyprogesterone	0.10	LOQ-2000
Progesterone	0.04	LOQ-1500

Table S1. Overview of limits of quantification (LOQ) and linear range of detection (in nM concentrations) for the steroid hormones measured by TurboFlow-LC-MS/MS in the clinically validated analysis packaged.
