# Supplementary material for: The effects of selected inhibitors on human fetal adrenal steroidogenesis differs under basal and ACTH-stimulated conditions
Source: BMC Med. 2021 Sep 8;19:204. doi: 10.1186/s12916-021-02080-8 (PMC8425147; doi:10.1186/s12916-021-02080-8)
Supplement: Supplementary file 2 — Additional file 2. Results of the dose-response of Abiraterone acetate treatment. This file shows ex vivo cultured HFAs steroid hormone levels following treatment with 1 μM and 10 μM Abiraterone acetate under basal conditions. [file 12916_2021_2080_MOESM2_ESM.pdf]

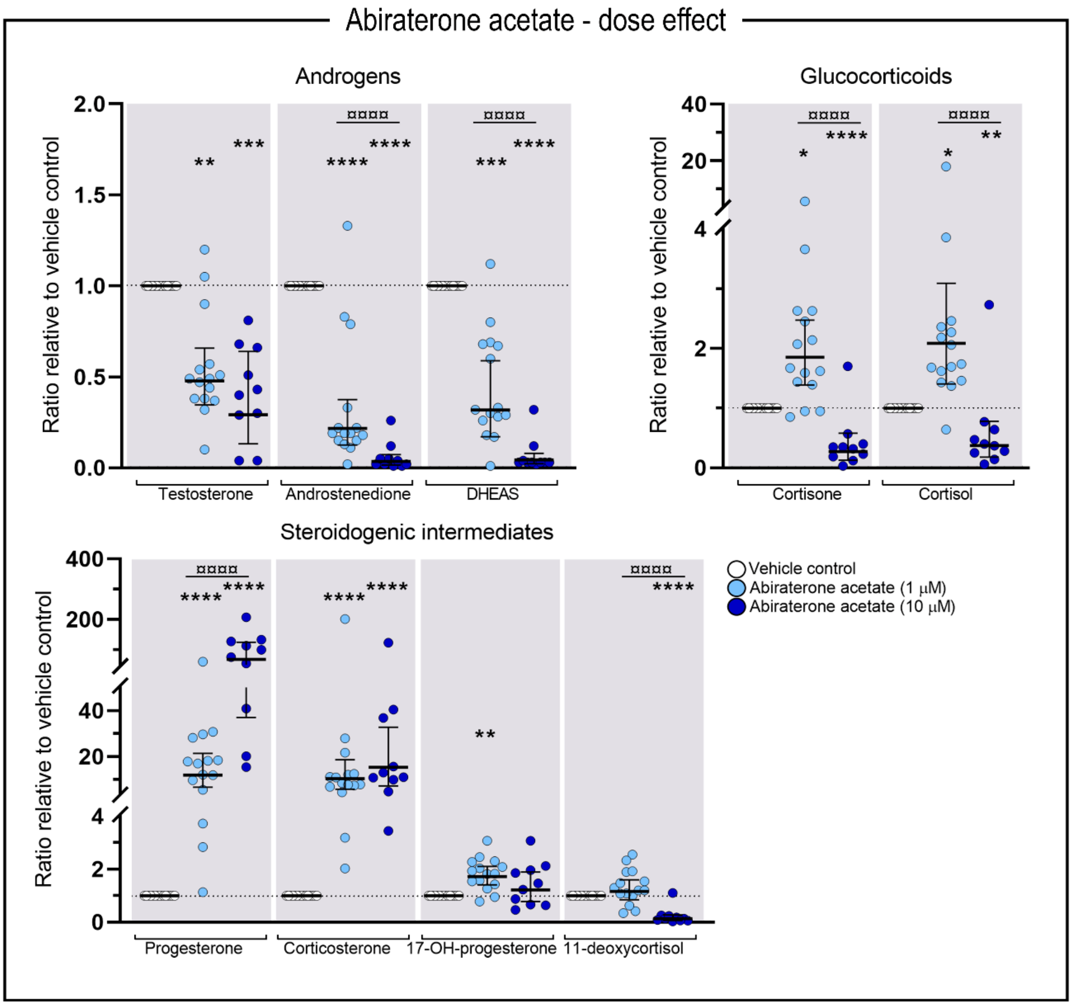

Figure S1. HFA tissue cultured *ex vivo* for 14 days treated with 1 µM and 10 µM Abiraterone acetate under basal conditions. Concentrations of secreted adrenal steroid metabolites were measured by LC-MS/MS in the collected culture media and are presented as a ratio relative to vehicle controls from the same HFA gland. Values represent geometric mean with 95% CI. Dose effects were examined in adrenal tissue originating from different fetuses. Treatment dose: 1 µM n = 15 (Note, 10/15 samples are also included in Figure 2); Treatment dose: 10 µM n = 10, with media pooled from 1-13 tissue fragments per treatment depending on the initial size of half of the adrenal gland. Significant difference compared with vehicle controls from the same fetus were based on ln-transformed data using ordinary one-way ANOVA followed by Tukey's multiple comparisons test. (*) indicate differences compared with vehicle controls, (¤) indicate differences compared with Abiraterone Acetate (1 µM) treatment. */¤p<0.05, **/¤¤p<0.01, ***/¤¤¤p<0.001, ****/¤¤¤¤P<0.0001. DHEAS, dehydroepiandrosterone-sulfate.
